# Case report: Short-course hypofractionated radiation therapy combined with immune checkpoint inhibitors for the treatment of advanced ovarian mucinous cystadenocarcinoma

**DOI:** 10.3389/fonc.2025.1430474

**Published:** 2025-01-20

**Authors:** Xinan Wan, Mingxing Fang, Le Yuan, Hang Zhang, Dan Wang

**Affiliations:** Department of Oncology, The Second People’s Hospital of Wuhu City, Wuhu, China

**Keywords:** ovarian mucinous cystadenocarcinoma, hypofractionated radiation therapy, PD-1 inhibitors, case report, multiple-line treatments

## Abstract

**Background:**

Ovarian mucinous cystadenocarcinoma is a rare subtype of ovarian epithelial carcinoma that is resistant to platinum-based chemotherapy and has a poor prognosis, and there is no standard treatment plan for patients for whom multiline treatment has failed.

**Case presentation:**

oma with FIGO stage IVB disease who was sequentially treated with paclitaxel liposomes+carboplatin, 5-Fu+CF+oxaliplatin, capecitabine+oxaliplatin, bevacizumab+FOLFOX4, S-1, and bevacizumab+oxaliplatin+raltitrexed chemotherapy. After the progression of the disease, a combination of short-course hypofractionated radiation therapy and immune checkpoint inhibitors was used. The radiotherapy target area was the metastatic lymph nodes in the right posterior part of the hepatic artery, with a radiation dose of 30 Gy/10 F. Camrelizumab, an immune checkpoint inhibitor, was administered intravenously every three weeks at a dose of 200 mg each time. The therapeutic effect was significant, with CA125 levels within the normal range. Metastatic lymph nodes disappeared from the abdominal cavity. The therapeutic effect achieved a complete response (CR). Currently, CA125 levels are within the normal range, and abdominal CT reveals no tumor recurrence or metastasis. The duration of response (DoR) reached over four years.

**Conclusion:**

Ovarian mucinous cystadenocarcinoma is a rare tumor with poor treatment efficacy and poor prognosis. Short-course hypofractionated radiation therapy combined with PD-1 inhibitors may be an effective and safe treatment strategy.

## Introduction

Multiple clinical studies have confirmed the effectiveness of radiotherapy combined with immunotherapy. The PEMBRO-RT study (1) compared pembrolizumab combined with stereotactic body radiotherapy (SBRT) and pembrolizumab in the treatment of advanced non-small cell lung cancer, and the results showed that the ORR of the combination group and monotherapy group at 12 weeks was 41% and 19%, respectively, with a median PFS of 6.4 months and 1.8 months, respectively. In the ComIT-1 study (2), atezolizumab+SBRT was applied as a second-line and posterior-line treatment for advanced NSCLC patients; four patients achieved a partial response (PR), eight patients had stable disease (SD), and 10 out of 15 PD-L1-negative patients survived for more than 18 months. The PACIFIC study (3–6) included nonsurgical stage III NSCLC patients. The combination group received durvalumab sequential treatment for 1 year after concurrent chemoradiotherapy, while the control group only received concurrent chemoradiotherapy. The PFS times of the combination group and the control group were 16.8 months and 5.6 months, respectively. The 3-year overall survival (OS) rates were 57% and 43.5%, respectively. The disease progression or mortality risk was reduced by 48% in the combination group, and there was no statistically significant difference in quality of life-related indicators between the two groups (7). Herein, we report a patient with advanced ovarian mucinous cystadenocarcinoma who achieved a clinical complete response after radiotherapy combined with immune checkpoint inhibitors.

## Case presentation

**Figure 1:**
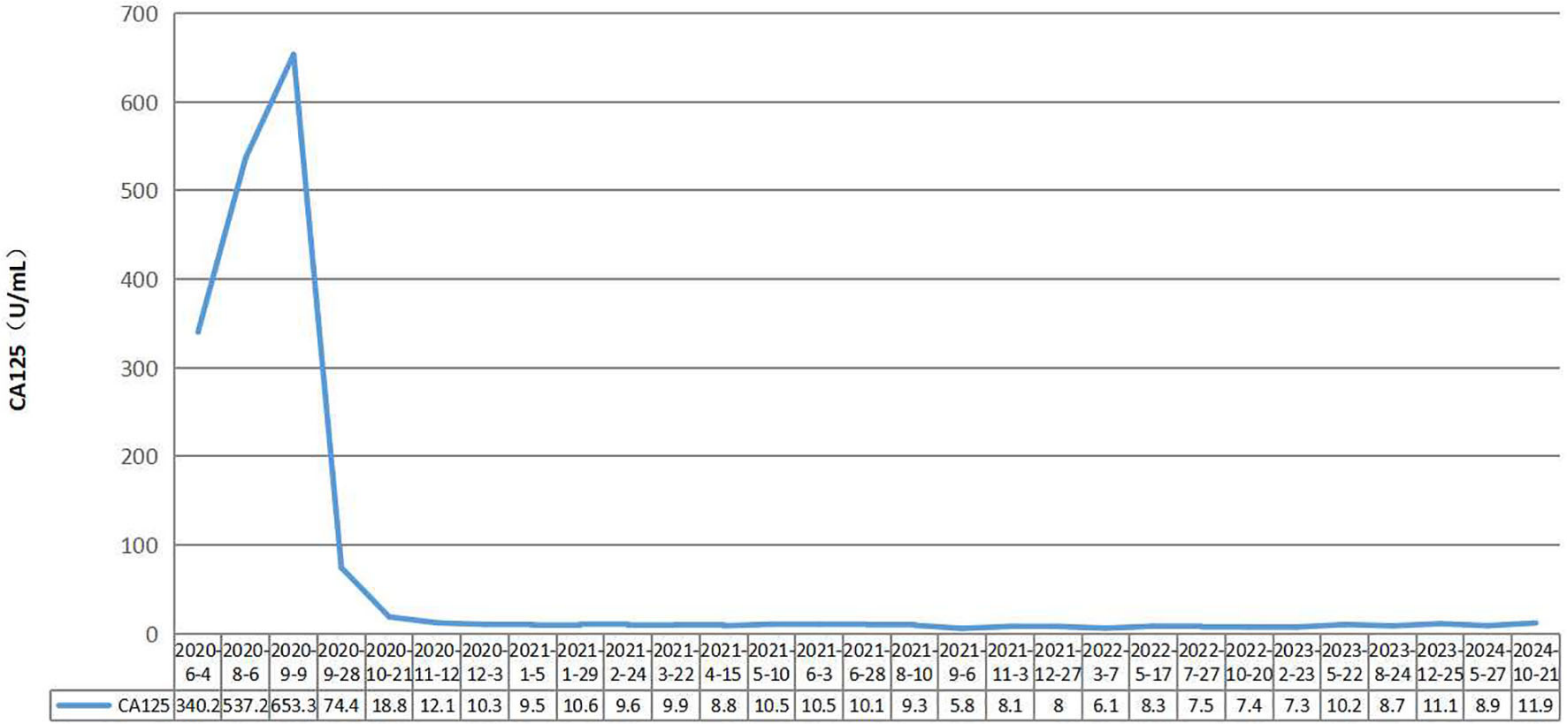
Changes of CA125 throughout the process of the treatment.

**Figure 2:**
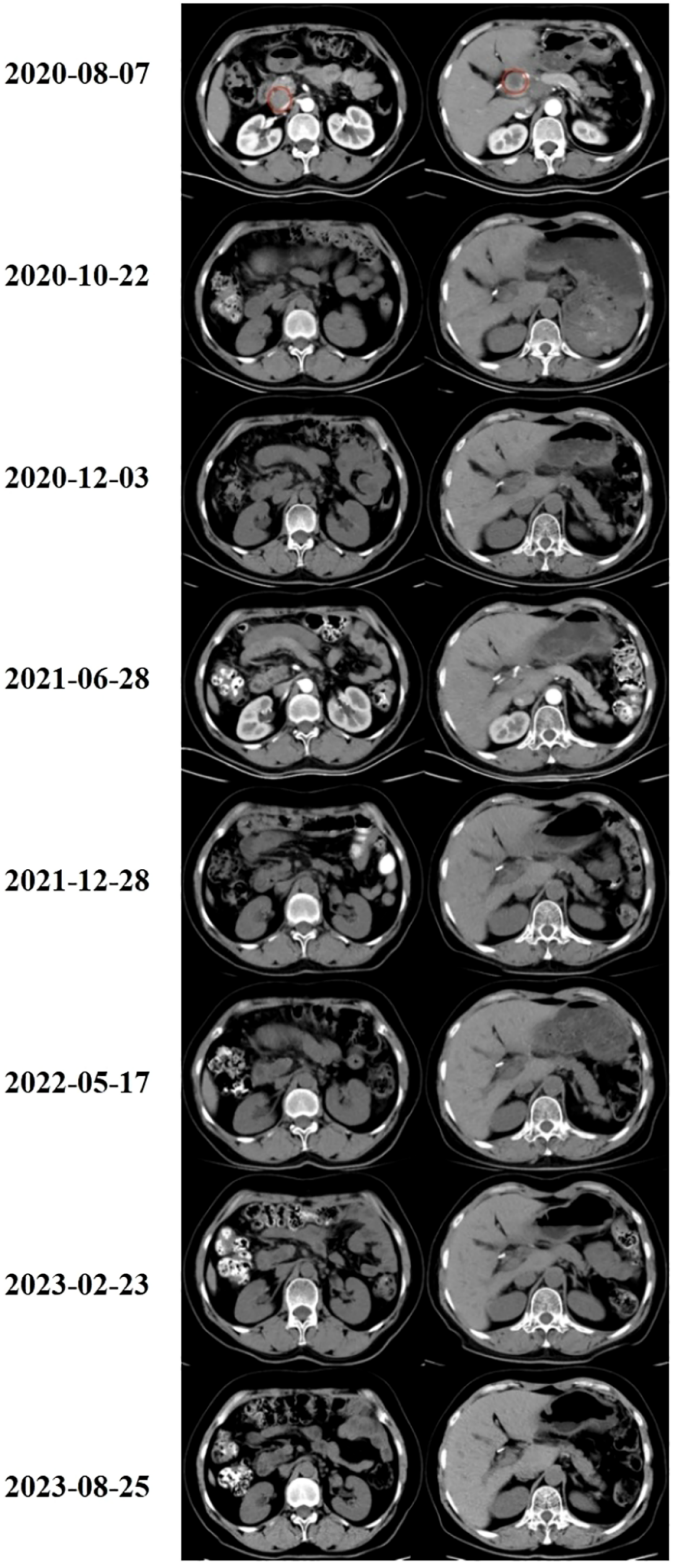
Abdominal CT scanning of the patient. (2020-08-07)The metastatic lymph nodes in the right posterior part of the hepatic artery were shown by CT before treatment. (2020-10-22 to 2023-08-25)After short-course hypofractionated radiation therapy combined with PD-1 inhibitors, CT revealed the disappearance of metastatic lymph nodes in the abdominal cavity. The therapeutic effect achieved a complete response.

A 56-year-old female patient with lower abdominal pain visited a local hospital on November 18, 2018. She is a Chinese with a height of 160cm and a weight of 68kg. PET-CT revealed a cystic-solid mass in the pelvic cavity, multiple low-density lesions in the spleen, nodules in the left cardiophrenic angle area, a lump in the hepatic hilar region, a cystic-solid mass around the hepatic flexure, and an abnormal increase in FDG metabolism. It is suspected that ovarian cystadenocarcinoma is accompanied by multiple metastases in the spleen, left cardiophrenic angle area, and abdominal cavity and pelvic effusion. On November 26, 2018, exploratory laparotomy, ovarian cancer cytoreductive surgery, paraaortic lymph node dissection, anterior sacral lymph node dissection, pelvic lymph node dissection, splenectomy, pancreatic head mass resection, omentectomy, and appendectomy were performed at the hospital. On December 3, 2018, postoperative pathology revealed moderate to poor differentiation of mucinous cystadenocarcinoma of the spleen, with a tumor size of approximately 9.0 cm×8.0 cm×6.0 cm. Lymphovascular invasion. There was no perineural invasion. Cancer tissue involvement can be observed in the fibers and muscle tissue of the posterior peritoneum. Cancer tissue involvement was observed in the greater omentum. There was no cancer involvement in the appendix. No cancer involvement was found in the uterine body, cervical canal, cervix, or left or right parametria. One gray white tissue sample (mass above the pancreatic head) approximately 7.0 cm×5.0 cm×4.5 cm in size was sent for examination, and microscopic examination revealed lymph node metastatic mucinous cystadenocarcinoma. Eight lymph nodes (adjacent to the abdominal aorta), 13 lymph nodes (in the left pelvic cavity), 21 lymph nodes (in the right pelvic cavity), and 6 lymph nodes (in the anterior sacral region) were sent for examination, totaling 48 nodes, and no cancer metastasis was found (0/48). According to postoperative pathology, the FIGO stage was stage IVB. Chemotherapy began on December 25, 2018. Due to tumor progression or intolerable adverse reactions, the treatment regimen was changed multiple times. Four courses of the paclitaxel liposome+carboplatin regimen, three courses of the 5-Fu+CF+oxaliplatin regimen, three courses of the capecitabine+oxaliplatin regimen, one course of the bevacizumab+FOLFOX4 regimen, one course of S-1 chemotherapy, and two courses of the bevacizumab+oxaliplatin+raltitrexed regimen were administered. On June 4th, 2020, the CA125 concentration increased to 340.2 U/mL; on August 6th, 2020, the CA125 concentration increased to 537.2 U/mL; and on August 7th, 2020, abdominal and pelvic contrast-enhanced CT revealed increased numbers of lymph nodes in the abdominal cavity. The disease progressed after multiline treatment, and there was no standard treatment plan to follow. Short-course hypofractionated radiation therapy combined with immune checkpoint inhibitors was administered. Starting on August 11, 2020, three-dimensional conformal radiation therapy with a radiation dose of 30 Gy/10 F was administered to the metastatic lymph nodes in the right posterior part of the hepatic artery. Starting on August 19, 2020, the PD-1 inhibitor camrelizumab was administered intravenously every three weeks at a dose of 200 mg each time. The therapeutic effect was significant. On September 28th, 2020, the CA125 concentration decreased to 74.4 U/mL, and on October 21, 2020, it decreased to 18.8 U/mL, falling within the normal range (**Figure 1**). On October 22, 2020, abdominal CT revealed the disappearance of metastatic lymph nodes in the abdominal cavity. The therapeutic effect achieved a complete response (CR) (**Figure 2**), and maintenance treatment with camrelizumab continued until July 28th, 2022. Currently, CA125 levels are within the normal range, and abdominal CT reveals no tumor recurrence or metastasis. The duration of response (DoR) reached over four years. During the course of treatment, the patient did not experience any treatment-related adverse events such as fatigue, bone marrow suppression, or impaired liver and kidney function, and had good tolerance.

## Discussion

Ovarian mucinous cystadenocarcinoma is a rare subtype of ovarian epithelial cancer that accounts for 3-5% of ovarian epithelial cancers (8). Traditionally, the treatment methods for ovarian mucinous cancer are similar to those for more common ovarian serous cancer. However, studies have shown (9, 10) that compared to other types of ovarian epithelial cancer, ovarian mucinous cystadenocarcinoma is resistant to platinum-based chemotherapy. The treatment for gastrointestinal cancer is another option, and these drugs include 5-fluorouracil, oxaliplatin, and capecitabine (11). In addition, bevacizumab may be beneficial for the treatment of recurrent or progressive ovarian mucinous cancer (12). The prognosis of ovarian mucinous cancer patients is poor, and the median overall survival of stage III/IV patients is less than 15 months (13).

Due to the rarity of ovarian mucinous cystadenocarcinoma, large-scale prospective studies on its treatment are rare, and there is an urgent need for new treatment methods. In this case, the patient underwent multiline chemotherapy, ranging from standard platinum-based chemotherapy to a gastrointestinal tumor chemotherapy regimen and bevacizumab, which had poor efficacy and a short response period. We used short-course hypofractionated radiation therapy combined with PD-1 inhibitors for treatment and achieved a complete response. Hypofractionated radiation therapy is a method of dividing the total radiation dose into larger doses, with fewer treatment sessions and a fractionated dose that is higher than the conventional dose of 2Gy. To date, the duration of response has been over four years. The reason for the better therapeutic effect may be that radiotherapy affects the tumor immune microenvironment, releases tumor-associated antigens and a series of immune stimulating factors, induces tumor-specific immune occurrence, and transforms immune “cold tumors” into “hot tumors” (14, 15). In addition, local radiotherapy can also cause tumor regression in nonirradiated distant areas, known as the abscopal effect of radiotherapy. The mechanism of the abscopal effect is very complex and is mainly mediated by the body’s antitumor immune system. However, abscopal effects are rare in clinical cases. In recent years, it has been found that radiotherapy combined with immune checkpoint inhibitors can increase the incidence of the abscopal effect. A study (16) revealed that high-dose radiation therapy combined with anti-PD-1/PD-L1 antibodies for the treatment of tumor-bearing mice induced an abscopal effect, inhibiting tumor growth in nonirradiated areas. Another study (17) reviewed 16 clinical trials that combined the CTLA-4 inhibitor ipilimumab and radiation therapy for metastatic melanoma patients and reported an abscopal effect in 26.5% of patients.

In conclusion, ovarian mucinous cystadenocarcinoma is relatively rare, and the current treatment plan mainly refers to ovarian serous cancer, but the treatment effect is poor. In this case, multiple-line treatments were ineffective, and the patient was subsequently treated with short-course hypofractionated radiation therapy combined with PD-1 inhibitors to achieve a complete response, which was maintained for over four years. We suggest that this method could be an effective and safe treatment strategy.

## Data Availability

The raw data supporting the conclusions of this article will be made available by the authors, without undue reservation.
